# Genito-Urinary and Syphilitic Diseases

**Published:** 1896-10

**Authors:** Byron H. Daggett

**Affiliations:** Buffalo, N. Y.


### Progress in Medical Science.

### GENITO-URINARY AND SYPHILITIC DISEASES.

Conducted by BYRON H. DAGGETT, M. D., Buffalo, N. Y.

### SOME POINTS IN THE TECHNIQUE OF LITHOLAPAXY.

CHISMORE (*Pacific Medical Journal* for July, 1896,) divides cases of litholapaxy into two classes: first, those in which the prostate gland is normal in size; second, those in which it is enlarged. In the first class the operator may confidently rely on clearing the bladder in one sitting; in the second class several crushings may be required. In cases where the prostate and urethra are normal, preliminary treatment is not necessary.

A stone having been discovered, located and its size determined, if there be no contra-indication, proceed at once to operate. Having placed the patient in the lithotomic posture, a catheter is passed and the bladder emptied. The catheter is withdrawn until the eye is just within the external end of the prostatic urethra. Then inject an ounce or an ounce and a half of a 4 per cent. solution of cocaine. Inject slowly, so that the solution laves and anesthetises the deep urethra as it passes onward into the bladder. The lithotrite should be very gently and slowly passed, without force.

Give a little time for relaxation if there be spasm of the constrictor muscle, and then tilt the beak of the instrument slightly upward, when it will slip into the bladder without the slightest pressure. If the stone be soft it is easily crushed by means of the hands alone ; if it be hard, the rack and pinion is brought into play. Bear in mind, the broken fragments usually gravitate to the locality originally occupied by the stone.

The operator should remember, if the beak of the lithotrite is reversed, that the line of the reflexion of the recto-vesicle fold of the peritoneum fixes in a measure that portion of the bladder to which it is attached, and he should be guarded lest he grasps a fold of the bladder in the jaws of his instrument. A knowledge of the anatomy and the *tactus eruditus* will serve to prevent injury. If the stone be small the operation may be completed without removing the instrument ; but if it is large, it is better and saves time to remove the lithotrite from time to time and aspirate through a tube as large as the urethra will admit. If the lithotrite becomes clogged, it may be cleared by quick, light strokes of the jaws.

Dr. Chismore states that unless the stone be very hard he always uses his own instrument, but if it be very hard a preliminary crushing is done with an instrument devised by the late Dr. Forbes, of Philadelphia, as this instrument is much stronger, and completes the operation with his own instrument, washing out the débris while the crushing is going on. When the bladder is empty the patient is sent home and directed to stay in bed till the soreness is gone.

Such an operation takes but a few minutes, can be safely done in the office, is almost devoid of danger and nearly painless ; but one assistant is needed and, if necessary, the operation may be done without an assistant. A second operation is rarely required, as the few grains of sand left are passed in urination.

The second class of cases present a very different aspect of affairs. " Catheter life " has usually begun and the urethra must be frequently invaded by an instrument following the operation ; therefore it is very necessary to avoid injuring the canal or greatly irritating it.

The topography of the interior of the bladder is decidedly changed and often forms pockets, which are quite out of reach of any lithotrite. In such a case the calculus, or its fragments, may be made to meet the instrument by means of a stream of water from the aspirator, passing out and in through the lithotrite, at the same time working the jaws to learn if the calculus has been brought within the grasp of the instrument. In cases of hypertrophy of the prostate the patient should be told that several crushings will probably be required to clear the bladder.

If after the preparation of the patient the stone cannot be caught, the jaws of the lithotrite are opened as widely as the bladder will admit, and aspiration is quickly and smartly compressed several times in succession, when the stone will frequently be found on gently closing the jaws. This procedure may be repeated as long as it is thought advisable.

If spasm, excessive pain, exhaustion or other untoward symptoms occur, the operation should be abandoned for the time being. In difficult cases, where the stone cannot be located by the Thompson searcher, it is useless to attempt to search with the lithotrite, and the procedure should be temporarily abandoned and another attempt made within a few days, when the stone may be readily found. It is better to stop too soon than to risk setting up inflammation by too prolonged manipulation. The number of sittings will vary with the varying difficulties presented in different cases as well as the skill and experience of the operator.

During the intervals Dr. Chismore avoids washing out the bladder, as he alleges that it is useless and dangerous ; if the urine becomes foul, irrigation will make it worse. Relief of these conditions will be obtained by removing the fragment which causes them. Dr. Chismore presents a new instrument for removing fragments in litholapaxy, for which the following claims are made : 1. A larger caliber of the catheter, circular in form, in the male bladder admitting a freer passage of fragments. 2. Merit as a searcher in cases of stones that evade the usual method of exploration. 3. Simplicity of construction and readiness with which it can be taken apart for cleaning. 4. Enabling the operator to proceed, after the first crushing and aspiration, uninterruptedly with the operation, diminishing the necessity of the repeated introduction and removal of different instruments, thus lessening the danger of injury to the deep urethra and shortening the time required. 5. Enabling the operator to avoid working with the beak reversed, thus avoiding the most dangerous region, the fundus of the bladder, the only part where it can be easily grasped by the lithotrite. Dr. Chismore reports fifty-eight cases of litholapaxy made by means of his instrument, which he says strengthens his claims for the same. He uses the instrument of Dr. Forbes for crushing the hard stones, and says that with these two instruments skilfully handled he fully believes it is possible to remove any stone that could be gotten by perineal section.

Dr. Chismore's instrument is manufactured by George Tieman & Co., 107 Park row, New York City, and it is illustrated in A system of genito-urinary diseases, syphilology and dermatology, by Prince A. Morrow, M. D.

Dr. Chismore recommends smearing the male blade of the lithotrite with Packer's tar soap. The wash bottle should be boiled in a strong solution of boric acid before and after each operation, which cleanses it thoroughly and preserves the rubber. During the operation two aspirators are very convenient: while one is being emptied the other is always ready for use.

He uses cocain in all operations for stones in which an anesthetic is needed. On a very few occasions there has been slight toxis, but never enough to cause suspension of the operation. Eight ounces of a four per cent. solution have been used in the course of three hours. Out of seventy-eight cases there have been but two deaths, neither one due to the operation. Dr. Chismore states that he had often been astonished at the very slight amount of distress resulting from even prolonged manipulations.

---

### DILATATION OF THE PREPUCE.

W. S. Stewart, in the Medical Council of Philadelphia, proposes dilatation of the prepuce as a substitute for circumcision. Dr. Stewart has devised a preputial dilator which consists of four bevel-edged blades attached to a curved forceps handle.

The blades are inserted into the preputial orifice, with care not to enter the urethral meatus; then by gradual pressure upon the handle the foreskin is stretched until it may be readily retracted over the glans. If there be adhesions to the glans, the mucous membrane should be dissected off with a blunt probe. The probe can usually be passed under the membrane to the sulcus behind the corona, which will aid the process.

The abstractor has found cases where the adhesions were so firm that a bistoury was also required to detach the membrane from the glans. The prepuce should be retracted every day and some bland unguent applied until the foreskin is relaxed and contracted. If a redundancy of tissue remain, it can be easily removed afterward. Circumcision is dreaded for the reason that it is a cutting procedure.

Dr. Stewart alleges that this method of dilatation has proved very satisfactory, doing away with the knife, drawing no blood, and there is no mutilation of the organ ; also, that if the operation be carefully and thoroughly done there will be no need of any other remedy.

